# Prognostic value of tertiary lymphoid structure and tumour infiltrating lymphocytes in oral squamous cell carcinoma

**DOI:** 10.1038/s41368-020-00092-3

**Published:** 2020-09-15

**Authors:** Qunxing Li, Xiangqi Liu, Dikan Wang, Yanqiong Wang, Huanzi Lu, Shuqiong Wen, Juan Fang, Bin Cheng, Zhi Wang

**Affiliations:** Hospital of Stomatology, Guanghua School of Stomatology, Guangdong Provincial Key Laboratory of Stomatology, Sun Yat-Sen University, Guangzhou, China

**Keywords:** Oral cancer detection, Prognostic markers, Cancer microenvironment

## Abstract

Tertiary lymphoid structures (TLS) are ectopic lymphoid structures in cancers that are largely associated with favourable prognosis. However, the prognostic value of TLSs in oral squamous cell carcinoma (OSCC) is largely unknown, and the association between tumour infiltrating lymphocytes (TILs) and TLSs has been rarely explored in OSCC. In this study, associated markers of TLS, including peripheral node address (PNAd) in high endothelial venules, CD20 in B cells and CD3 in T cells, were examined in 168 OSCC patients, and survival analysis was performed between TLS-positive and TLS-negative cohorts. We detected the presence of TILs by staining CD8+ cytotoxic T cells and CD57+ NK cells as well. TLSs appeared as highly organized structures in 45 (26.8%) cases. TLS-positive patients had a better 5-year overall survival (OS) rate (88.9% vs. 56.1%, *P* < 0.001) and relapse-free survival (RFS) rate (88.9% vs. 63.4%, *P* = 0.002). Moreover, the presence of TLS was an independent prognostic factor for both the 5-year OS rate (hazard ratio [HR] = 3.784; 95% confidence interval [CI], 1.498–9.562) and RFS rate (HR = 3.296; 95% CI, 1.279–8.490) in multivariate analysis. Furthermore, a higher density of CD8+ T cells and CD57+ NK cells was found in TLS-positive sections than in TLS-negative counterparts (*P* < 0.001), and their combination provided a higher predictive accuracy (AUC = 0.730; 95% CI, 0.654–0.805). In conclusion, our results suggest that TLS is an independent positive prognostic factor for OSCC patients. These findings provide a theoretical basis for the future diagnostic and therapeutic value of TLSs in OSCC treatment.

## Introduction

Oral squamous cell carcinoma (OSCC) is a major type of head and neck cancer, accounting for ~2%–4% of all incident cancer cases per year.^1^ Because of the high rate of metastasis to cervical lymph nodes at early stages, OSCC is regarded as an aggressive type of cancer. Although the treatment of OSCC has evolved from surgical resection to surgery-based comprehensive therapy including radiation and chemotherapy in the past few decades,^2^ the current average 5-year overall survival (OS) rate is only ~65% in the world.^3^ In recent years, immune checkpoint blockade therapy, which reinvigorates antitumour CD8+ T cells by blocking programmed cell death protein 1 (PD-1) or programmed cell death ligand 1 (PD-L1), has provided a novel approach for cancer therapy;^4^ however, the response rate of OSCC is only ~13%.^5^ Therefore, given the poor prognosis of OSCC, it is imperative to select and identify a marker with good diagnostic and prognostic value. However, until now, no specific markers have met the requirements for OSCC treatment.

Tertiary lymphoid structures (TLSs) are ectopic, vascularized lymphoid structures that can be found in inflamed or tumour tissues.^6^ Similar to secondary lymph organs (SLOs), including lymph nodes, typical TLSs are composed of B-cell zones containing active germinal centres, and surrounding T-cell zones that contain various types of dendritic cells (DCs), T cells and high endothelial venules (HEVs).^7^ However, unlike lymph nodes, TLSs lack fibrous capsules outside and are directly exposed to the tumour microenvironment (TME), which tends to be more easily stimulated by tumour antigens and cytokines.^8^ It is believed that the formation of TLSs results from long-term exposure to inflammatory cytokines and chemokines in infectious diseases, autoimmune diseases or tumours and TLSs are reported to play important roles in inducing effector functions, affinity maturation, antibody generation, class switching and clonal expansion.^9^ Thus, TLSs might be involved in the antitumour immune response.^6,9,10^

Currently, studies of TLSs are mostly focused on non-small-cell lung cancer, colorectal cancer, melanoma and breast cancer.^11–16^ Increasing studies have shown that the presence of TLSs is associated with better prognosis, and TLSs have become a predictive marker for the favourable efficacy of immune checkpoint blockade therapy, whereas other studies have shown adverse results in cancer.^17,18^ However, the prognostic value of TLS in OSCC is largely unknown. By using immunohistochemical staining for 80 OSCC tumour samples, Wirsing et al.^19^ found that the presence of TLS was associated with a higher disease-specific survival rate (DSS) in univariate analysis, while no significant result was found in multivariate analysis. Given the small number of cases in the previous study, more evidence is needed to evaluate the prognostic value of TLS for OSCC.

Tumour infiltrating lymphocytes (TILs) are a select population of lymphocytes in tumours with higher specific immunological reactivity against tumour cells than noninfiltrating lymphocytes.^20^ It has been reported in various types of cancers that higher TIL levels in tumours are associated with better prognosis.^20^ Several studies have shown a positive correlation between TIL and TLS levels in breast cancer, colorectal cancer and ovarian cancers.^15,21,22^ We previously identified that a high density of CD8+ and CD57+ TILs in the tumour stroma independently predicted the prolonged overall survival of OSCC patients.^23^ To evaluate the prognostic value of TLS on OSCC patient survival, we detected and classified TLS in a large cohort of 168 OSCC patients by using immunohistochemical staining of PNAd, CD20 and CD3 for HEV, B cells and T cells, respectively. Then, we analysed the prognostic value of TLS in OSCC. Furthermore, we stained cells for CD8 and CD57 to analyse the association between TIL and TLS in OSCC and their relationship with prognosis. Our findings provide a theoretical basis for the diagnostic and therapeutic value of TLS in OSCC treatment.

## Results

### TLSs are highly organized structures in OSCC and peritumoural dysplasia

**Fig. 1:**
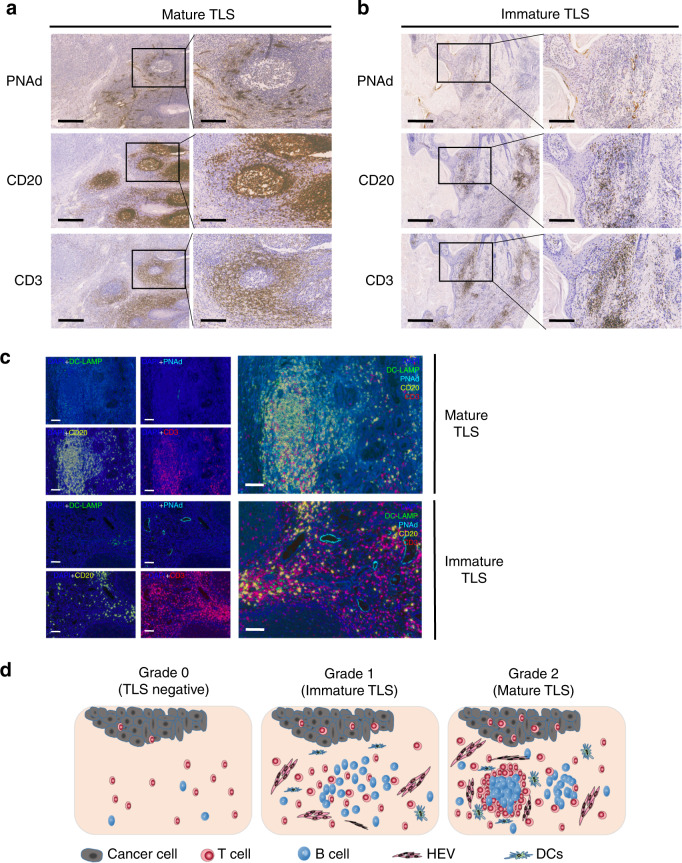
TLSs are highly organized structures in OSCC tissues and appear as clusters of B-cell follicles surrounded by T-cell zones and HEVs. **a** Mature TLS, similar to lymph node; CD20+ B cells form a follicular structure. The CD3+ T-cell zone is located near the follicle, and HEV are diffusely distributed around the follicle. **b** Immature TLS; CD20+ B cells do not form any follicular structure within the PNAd+ HEV area. CD3+ T cells were more diffusely distributed than mature TLSs. **c** Representative multiplex IHC images showing immune cells of immature TLS and mature TLS samples by simultaneous staining of B cells (CD20, yellow), T cells (CD3, red), mature dendritic cells (DC-LAMP, green), high endothelial venules (PNAd, cyan) and the nuclear stain DAPI (blue) (×200, original magnification). **d** TLS grading in OSCC tissues. Grade 0, no TLS present; grade 1, immature TLS; grade 2, mature TLS. Scale bar, 500 μm in “4X” pictures and 200 μm in “10X” pictures

We investigated the presence of TLSs in tumour specimens from 168 patients with OSCCs using immunohistochemistry. The patient characteristics are summarized in Table S1. In total, TLS was defined in 45/168 (26.8%) OSCC samples. The correlation analyses between TLS and the clinicopathological parameters are listed in Table 1. TLS typically appear as clusters of T-cell zones, B-cell zones and HEVs. Similar to lymph nodes, CD20+ B cells form a follicular structure in many cases. The CD3+ T-cell zone is located near the follicle, and HEVs are diffusely distributed around the follicle. We further classified TLS into mature TLS (17/168, 10.1%) and immature TLS (28/168, 16.7%). It is categorized as a mature TLS if CD20+ B cells gather into the follicular structure, regardless of the density of the TLS. The samples that displayed scattered T cells and B cells were classified as immature TLSs. In our study, nearly all the mature TLS samples (16/17, 94.1%) were located in the stroma of the tumour margin, while some immature TLS samples (13/28, 46.4%) displayed intratumoural TLS (Fig. 1a, b).

**Table 1:** Correlation analyses between TLS and the clinicopathological parameters

Variables	TLS negative (*n* = 123)	TLS positive (*n* = 45)	*P* value
Gender			
Male	89	31	0.66
Female	34	14	
Age			
≤57	56	23	0.522
>57	67	22	
Smoking			
Current	60	24	0.602
Never or Former	63	21	
Alcohol			
Current	56	21	0.896
Never or Former	67	24	
Tumour site			
Tongue	52	21	0.665
Bucca	28	11	
Gingiva	30	7	
Others	13	6	
Differentiation			
High	71	32	0.116
Medium/Low	52	13	
T stage			
T1/T2	70	32	0.096
T3/T4	53	13	
Nodal invasion			
Negative	59	31	0.016*
Positive	64	14	
CD8			
High	34	37	<0.001*
Low	89	8	
CD57			
High	39	33	<0.001*
Low	84	12	

*TLS* tertiary lymphoid structure

*P < 0.05

To further identify the TLS and understand the spatial distribution among the various immune cells, we performed multiplex immunohistochemistry (mIHC) on these samples. We found CD3+ T cells, CD20+ B cells, PNAd+ HEV and DC-LAMP + (LAMP3) DCs expressed on both immature and mature TLSs (Fig. 1c). Moreover, follicular structure centred on B cells was formed in mature TLSs, and many T cells were aggregated around and within the follicles. In addition, LAMP3 + DCs and PNAd+ HEV were distributed at the edge of the follicle. In contrast, T cells and B cells were scattered diffusely in immature TLSs without follicular structure formation, but there seemed to be more abundant DCs and HEVs. According to the density of CD20+ B cells and the formation of follicular structure in TLS, we classified it into three grades. Grade 0 represents TLS-negative, grade 1 refers to immature TLS and grade 2 is mature TLS (Fig. 1d). These results further revealed the distribution of immune cells in different types of TLSs, which may uncover the deeper interaction between different types of immune cells.

**Fig. 2:**
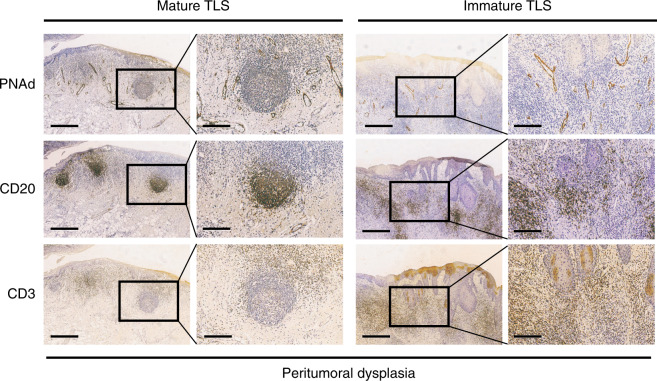
Mature and immature TLSs are also located in OSCC peritumoural dysplasia tissues. The structure is similar to those located in the cancer nests. Scale bar, 500 μm in “4X” pictures and 200 μm in “10X” pictures

As TLS formation is a dynamic process, we next determined whether TLSs were present in the early stage of tumorigenesis. Therefore, we further stained these markers in the peritumoural tissues ~5 mm outside the tumour margin, which were matched with the 45 TLS-positive tumour tissues. To our surprise, TLS staining was 73.3% positive (33/45) in the dysplasia area (Fig. 2). Moreover, both mature and immature TLSs existed in these samples (Fig. 2). These results indicated that TLS formation might start in the preliminary stage of malignant transformation.

### TLS was an independent prognostic factor for 5-year OS and RFS

**Fig. 3:**
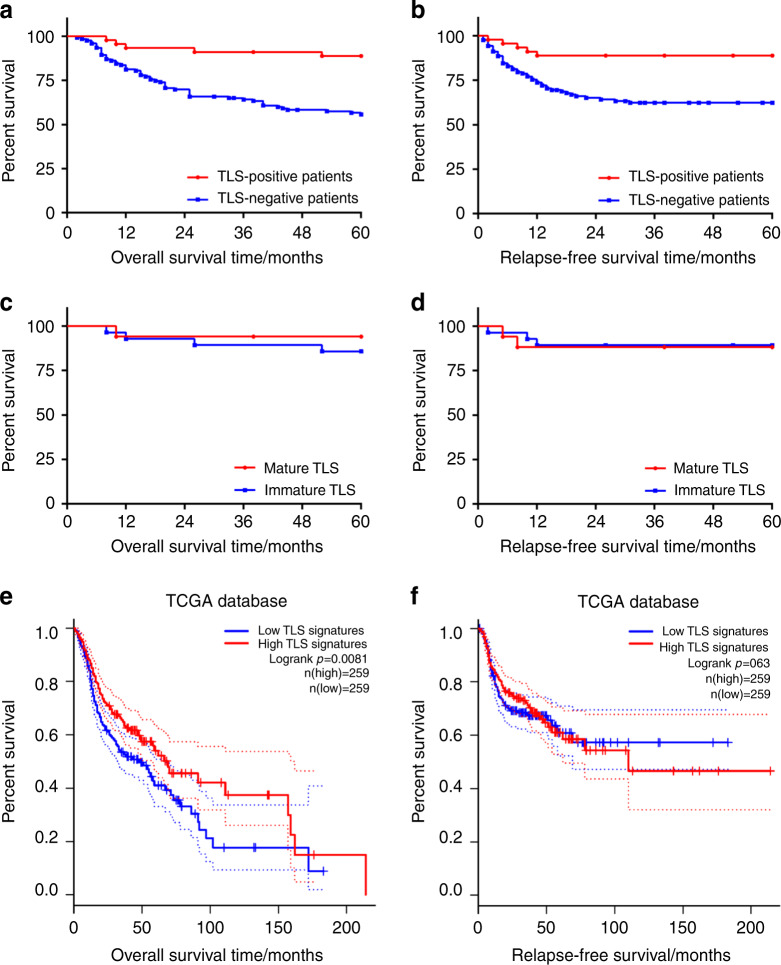
The presence of TLS could be a good prognostic factor in OSCC tissues. **a**, **b** Graphs showing the 5-year survival rates of TLS in 168 OSCC patients using the Kaplan–Meier method and log-rank test. **a** TLS-positive patients had a higher 5-year OS rate than TLS-negative patients (88.9% vs. 56.1%, *P* < 0.001). **b** TLS-positive patients had a higher 5-year RFS rate than TLS-negative patients (88.9% vs. 63.4%, *P* = 0.002). **c**, **d** The outcome of different subtypes of TLS in 45 TLS-positive OSCC patients using the Kaplan–Meier method and log-rank test. There were no significant differences in the 5-year OS and RFS rates between mature TLS and immature TLS in TLS-positive OSCC patients (94.1% vs. 85.7%, *P* = 0.411 and 88.2% vs. 89.3%, *P* = 0.896, respectively). **e**, **f** TCGA database showed that high TLS signature predicted better survival than low TLS signature in overall survival rate but no significance in relapse-free survival rate in head and neck cancer (*P* = 0.0081 and 0.63, respectively)

Next, we investigated the prognostic value of various clinicopathological variables in our cohort by univariate analysis using the log-rank test (Table S2). Among these variables, tumour differentiation, tumour stage, nodal invasion and TLS-positive tumours indicated a trend towards improved 5-year OS and RFS. TLS was significantly associated with a better 5-year OS rate (88.9% vs. 56.1% in the TLS-negative group; *N* = 168; *P* < 0.001) (Fig. 3a). Furthermore, TLS was also associated with a significantly better 5-year RFS rate (88.9% vs. 63.4% in the TLS-negative group; *N* = 168; *P* = 0.002) (Fig. 3b). On the other hand, we evaluated the survival of head and neck cancer in the TCGA database based on the gene signature of TLS.^16^ Consistent with our data, the overall survival rate of the high TLS signature group showed superior outcomes than the low TLS signature group (*P* = 0.008 1). However, there was no significant difference in the RFS rate (*P* = 0.63, Fig. 3e, f).

Variables that showed statistically significant associations with 5-year OS and RFS rates in the univariate analyses were entered into multivariate Cox regression analyses (Table 2). In multivariate analyses, TLS and CD8 expression were independent prognostic factors for both the 5-year OS rate (HR = 3.784, 95% CI, 1.498–9.562; HR = 2.301; 95% CI, 1.237–4.281) and RFS rate (HR = 3.296, 95% CI, 1.279–8.490; HR = 2.467, 95% CI, 1.178–4.731). Nodal invasion and CD57 expression were independent prognostic factors for 5-year OS (HR = 2.422, 95% CI, 1.365–4.299; HR = 1.579, 95% CI, 1.190–3.154). T stage was an independent prognostic factor for 5-year RFS (HR = 3.101, 95% CI, 1.656–5.808).

**Table 2:** Multivariate analysis of 5-year OS and RFS based on the Cox’s proportional hazards model

Variables	5-year OS/%		5-year RFS/%	
	HR (95% CI)	*P*	HR (95% CI)	*P*
Differentiation				
High	Reference	0.221	Reference	0.632
Medium/Low	1.399 (0.818–2.392)		1.155 (0.640–2.088)	
T stage				
T1/T2	Reference	0.158	Reference	0.001*
T3/T4	1.399 (0.858–2.570)		3.101 (1.656–5.808)	
Nodal invasion				
Negative	Reference	0.003*	Reference	0.588
Positive	2.422 (1.365–4.299)		1.178 (0.651–2.133)	
TLS				
Positive	Reference	0.005*	Reference	0.014*
Negative	3.784 (1.498–9.562)		3.296 (1.279–8.490)	
CD8				
High	Reference		Reference	
Low	2.301 (1.237–4.281)	0.009*	2.467 (1.178–4.731)	0.017*
CD57				
High	Reference		Reference	
Low	1.579 (1.190–3.154)	0.016*	1.093 (0.553–2.158)	0.399

*TLS* tertiary lymphoid structure; *OS* overall survival; *RFS* relapse-free survival; *HR* hazard ratio; *95% CI*, 95% confidence interval

*P < 0.05

However, when comparing the survival outcomes of immature TLS (*n* = 28) with mature TLS (*n* = 17), the results showed no significant differences for 5-year OS and RFS rates (94.1% vs. 85.7%, *P* = 0.411 and 88.2% vs. 89.3%, *P* = 0.896, respectively) (Fig. 3c, d). Then, we took another three variables from Table S2 as the candidate risk factors for TLS-positive patients and two new candidate factors for univariate and multivariate 5-year overall survival analysis, which is shown in Table S3. Nodal invasion was an independent prognostic factor for the 5-year OS rate (HR = 16.361; 95% CI, 1.314–203.793, respectively), while the other three candidate factors (differentiation, T stage and subtype of TLS) were not significantly different in our cohort.

### TLS correlates with high infiltration of CD8+ T cells and CD57+ NK cells

**Fig. 4:**
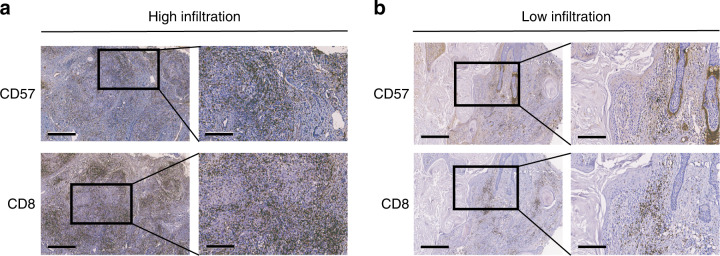
The distribution of CD8+ and CD57+ immune cells in OSCC tissues. **a** High infiltration of CD8+ and CD57+ immune cells in OSCC tissues. **b** Low infiltration of CD8+ and CD57+ immune cells in OSCC tissues. Scale bar, 500 μm in “4X” pictures and 200 μm in “10X” pictures

Considering that the antitumour response in the tumour environment mainly depends on cytotoxic T cells and NK cells, we next explored the relationship between TLS and tumour infiltrating cytotoxic immune cells.^24–26^ Positively stained immune cells demonstrated brown granules on the membrane. CD8 and CD57 expression in the high and low infiltrated groups is shown in Fig. 4a, b. The relationships between TLS and the density of immune cells are shown in Table S4. In the whole series, CD57 expression and CD8 expression were both positively associated with high TLS grade.

### TLS combined with TIL had high predictive accuracy on 5-year OS

**Fig. 5:**
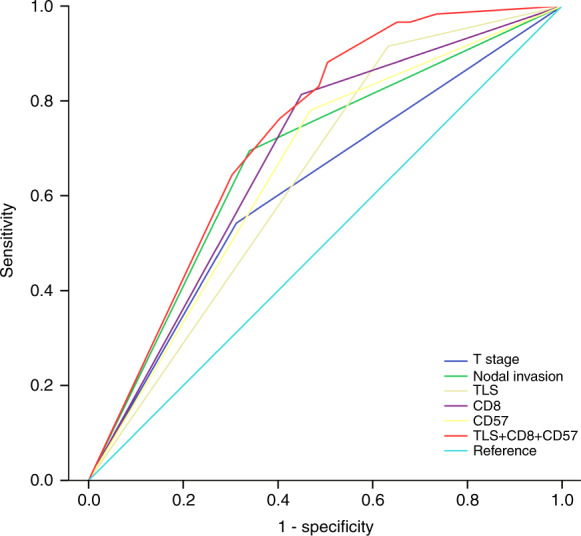
TLS combined with TIL had high predictive accuracy for 5-year OS. ROC curves indicating the predictive accuracy, sensitivity and specificity of each potential parameter. The AUCs of TLS, CD8 and CD57 were 0.641 (95% CI 0.558–0.725), 0.682 (95% CI 0.599–0.765) and 0.656 (95% CI 0.571–0.741, respectively), but TLS combined with CD8+ T cells and CD57+ NK cells provided the highest predictive accuracy (AUC = 0.730; 95% CI, 0.654–0.805)

To determine the predictive accuracy of TLS and TIL on 5-year OS, we performed ROC curve analyses. As shown in Fig. 5 and Table S5, T stage, nodal invasion, TLS, CD8 and CD57 had similar predictive accuracies (*P* < 0.05), but TLS combined with CD8+ T cells and CD57+ NK cells provided the highest predictive accuracy (AUC = 0.730; 95% CI, 0.654–0.805).

## Discussion

In this study, we evaluated the prognostic value of TLS in a large OSCC cohort. In general, the presence of TLS was an independent prognostic factor of the 5-year OS rate and RFS. High density CD8+ and CD57+ immune cell staining was positively associated with TLS positivity grading in OSCC. TLS combined with CD8+ T cells and CD57+ cells provided higher predictive accuracy. To the best of our knowledge, this is the first report of a large OSCC cohort for the evaluation of TLS and its prognostic value alone and in combination with TIL in OSCC.

Many studies have shown that TLS is associated with higher overall survival rates and remains an independent prognostic marker in colorectal cancer, breast cancer and pancreatic cancer.^15,27–29^ Consistent with their results, our cohort study revealed that the existence of TLS is associated with a superior prognosis, which indicated that TLS might induce a systemic long-lasting antitumour response in TME because TLS increased activated T cell and effector memory B-cell phenotype by upregulating a set of genes.^30–32^

To further explore different subtypes of TLSs, previous studies have divided TLSs into mature and immature TLSs or classical or nonclassical TLSs based on the density of CD20+ B cells and the formation of follicular structures. Consistent with previous studies, we found that in OSCC, mature or classical TLS typically comprise aggregates of B cells in a meshwork of follicular dendritic cells (FDCs) that are then surrounded by T cells as well as specialized blood vessels,^19,33,34^ whereas immature or nonclassical TLS is not typical. In our study, we discovered that both mature and immature TLS were present in the TME, but no significant difference was found when comparing the survival rate of OSCC patients between the mature and immature TLS groups.

Some studies found that TLS was more common in early-stage cancer, which implied that TLS formation might start in the initial stage of tumorigenesis.^13,35,36^ We found similar results in our cohort, although the difference between early-stage cancer and advanced cancer was not statistically significant. Interestingly, we also found TLS in 33 peritumoural dysplasia tissues of TLS-positive patients. Therefore, it is possible that at the early stage of OSCC, the local immune system may respond to antigen-stimulated immune cells, resulting in the formation of TLSs. Moreover, Workel et al.^37^ revealed that CXCL13 + CD103 + CD8+ TILs could also mediate B-cell recruitment and TLS formation in cancers.

As mentioned above, TLS is a lymphoid structure containing immune cells and vascular tissues, but the markers for defining TLS varied largely depending on the preference of the researchers and the composition of the TLS. For OSCC, a previous study adopted CD3 and CD20 as the core markers, while another study added CD21 as the marker of follicular DCs in the TLS.^19,38^ In the present study, we adopted PNAd, the specific marker for HEV, as the core marker in the TLS. HEVs, defined as the specific vessels in lymph nodes, are critical for the formation of TLSs and recruitment of naive B cells and T cells from blood to lymphatic tissues through bonding to L-selectin on the surface of naive lymphocytes and activation of B cells and T cells, which might help eliminate tumours.^9,39,40^ In the present study, we found that PNAd+ HEV is an indispensable subpopulation surrounding the TLS and is significantly associated with the prognosis of OSCC patients. Therefore, PNAd+ HEV is critical for TLS in OSCC with good identification and prognostic value.

TILs are a cluster of immune cells infiltrating the TME, and TILs have been observed to indicate a positive prognosis in our previous and other studies.^20,21,23,41^ However, no studies have shown whether TILs are correlated with TLS in OSCC patients until now. In the present study, high CD57+ and CD8+ immune cells were significantly associated with TLS grading in OSCC patients. NK cells can be recruited to tumour sites by chemokines, such as CXCL8 and CX3CL1.^42^ A recent single-cell sequencing study in hepatocellular carcinoma revealed that LAMP3 + DCs interacted with NK cells via NECTIN2-CD226.^43^ Moreover, activated DCs and T cells in TLSs can enhance NK cell proliferation through the production of cytokines such as IL-2, IL-15, IL-12, IL-18 and IFNα/β.^44–46^ In addition, patients with coexisting TLSs, CD57+ NK cells and CD8+ T cells seemed to have a better prognosis, which reminds us that TLSs might be a source of TILs and that TLSs might interrupt tumour progression by increasing TIL density. On the other hand, recent studies have shown that TLSs might have a vital role in sustaining the antitumour response by immune checkpoint blockade therapies.^16,32,47^ These findings will open a new avenue for therapeutic strategies that aim to increase TLS formation and enhance the function of TILs.

Conclusively, our findings indicate that the presence of TLSs, which are positively associated with TIL density in the TME, can be an independent positive prognostic marker for OSCC patients. Moreover, although TILs are associated with TLSs and their coexpression showed higher predictive accuracy in OSCC patients, the precise antitumour mechanism by TLS and the relationship with TILs in OSCC still need to be identified in further studies.

## Materials and methods

### Collection of clinical samples

In this study, the REMARK recommendations were broadly followed for tumour marker prognostic studies.^29^ A total of 168 consecutive patients with histologically verified primary OSCC from January 2007 to December 2010 were selected from the archives of the Guanghua School of Stomatology, Hospital of Stomatology, Sun Yan-sen University. The last day of follow-up was January 1, 2016. All patients had a close follow-up every 3 months during the first 5 years and then every 6 months 5 years later. Patients were followed until the closing date of the study or death, whichever came first.

The specimens were formalin-fixed, paraffin-embedded (FFPE) tumour resections from the tongue, gingiva, buccal mucosa, floor of the mouth and hard palate. Specimens from the base of the tongue were excluded. TNM classification was determined by the Union for International Cancer Control 2010 standard (UICC 2010). Pathological examination was performed by two independent pathologists. The use of human samples for this study was approved by the ethics committee of Sun Yat-sen University and was conducted according to the STROBE (Strengthening the Reporting of Observational Studies in Epidemiology) statement. All clinical data were kept anonymous.

### Immunohistochemistry

Four-micrometre sections of FFPE tissue from patients with OSCC were subjected to immunohistochemical staining. Before staining, all specimens were incubated at 60 °C for 2 h. Then, the samples were deparaffinized in xylene, rehydrated in graded alcohol baths, and incubated in 3% H_2_O_2_ for 20 min. Heat-induced antigen retrieval was performed in 0.01 M sodium citrate buffer at pH 6.0. Prior to antibody incubation, the slides were placed in a microwave and heated for 10 min at low temperature for antigen retrieval. Specimens were then incubated in 3% BSA for 30 min with the following primary antibodies: rabbit anti-CD20 (1:50, ab9475, Abcam, Cambridge, UK), rabbit anti-CD3 (MAB-0740, MXB Biotec, Fuzhou, China) and rat anti-PNAd (1:100, 120802, BioLegend, San Diego, CA, USA) overnight at 4 °C. Then, the cells were incubated with the secondary antibody at 37 °C for 30 min, stained with DAB for 3–8 min according to the primary antibody, and counterstained with haematoxylin.

### Multiplex immunohistochemistry

For TLS mIHC staining, 4-μm thick sections of the FFPE tissue were stained with the Opal 7-colour fluorescent IHC Kit (NEL811001KT, PerkinElmer, Massachusetts, USA). First, an initial deparaffinization procedure was performed on all slides, followed by 20 min of 10% formalin fixation and 15 min of Tris-EDTA (pH = 9.0) antigen retrieval under high temperature and high pressure. Afterwards, the slides were incubated with primary antibody, secondary-HRP and Opal TSA dyes for 16 h (4 °C), 10 min (RT) and 20 min (RT), respectively. Subsequently, the other rounds of staining consisted of antigen retrieval, blocking, primary antibody, secondary-HRP antibody and Opal TSA dyes. The following Opal detection fluorophores were used: CD20-Opal 690, CD3-Opal 620, PNAd-Opal 570, DC-LAMP (1:100, DDX0191P-100, Novus, Colorado, USA)-Opal 520. DAPI was used for nuclear counterstaining. The slides were finally mounted with Antifade Reagent (AR1109, BOSTER, Wuhan, China). The Vectra 3.0 imaging system and inForm image analysis software (Vectra 3.0; Perkin Elmer) were used to capture the immunofluorescence (IF) images and identify all markers of interest.

### Immunohistochemical evaluation

We used the following methods for TLS identification. First, the tissue sections were immunohistochemically stained for the HEV marker PNAd, the B-cell marker CD20 and the T-cell marker CD3 on consecutive sections. Samples had HEVs, and one or several accumulations of T cells around CD20-positive B cells were defined as TLS-positive. Second, according to the density of CD20+ B cells and the formation of follicular structure, we classified OSCC TLS into three categories: grade 0, no TLS present; grade 1, immature TLS; grade 2, mature TLS. We then calculated the area ratio of total OSCC tissue to the total TLS. TLS evaluation was blinded judging by two observers. If their judgement of TLS grading to the same case was different, the observers discussed the reasons for the difference and performed regrading. In the end, the results were checked by two additional pathologists from the pathology department in our hospital.

For TIL staining and detection, based on our previous work,^23^ we stained with cytotoxic T lymphocyte marker CD8 and natural killer cell marker CD57 antibodies. Briefly, 10 representative fields of the tumour tissue (×40 objective) were assessed by ImageJ software (V1.8.0.112, NIH). The total number of each immune cell type was counted in the tumour stroma, excluding those within tumour nests. The average number of 10 fields was calculated as the final density of each section (cells per field).

### Statistical analysis

All statistical analyses were performed using SPSS software (22.0, IBM). The Chi-square test was used to compare TLS expression and clinicopathological parameters. The 5-year OS rate and 5-year RFS rate were evaluated using the Kaplan–Meier method and log-rank test. The Cox proportional hazards model was used to estimate the independent prognostic factors for OS and RFS. *P* values < 0.05 were considered statistically significant. The area under the curve (AUC) and receiver operating characteristic (ROC) curve were used to evaluate and compare the prognostic value of TLS and TIL.

## Supplementary information

Table S1

Table S2

Table S3

Table S4

Table S5
