# Supplementary material for: Prognostic value of tertiary lymphoid structure and tumour infiltrating lymphocytes in oral squamous cell carcinoma
Source: Int J Oral Sci. 2020 Sep 15;12:24. doi: 10.1038/s41368-020-00092-3 (PMC7493903; doi:10.1038/s41368-020-00092-3)
Supplement: Supplementary file 1 — Table S1 [file 41368_2020_92_MOESM1_ESM.docx]

**Table S1.** Clinicopathological characteristics of OSCC patients

| Variables | | Number | Percentage (%) |
| --- | --- | --- | --- |
| Age | Mean (range) | 57 (24-83) |  |
|  | 57 | 79 | 47.02% |
|  | > 57 | 89 | 52.98% |
| Gender | Male | 120 | 71.43% |
|  | Female | 48 | 28.57% |
| Smoking | Current | 84 | 50.00% |
|  | Never or Former | 84 | 50.00% |
| Alcohol | Current | 77 | 45.83% |
|  | Never or Former | 91 | 54.17% |
| Tumor site | Tongue | 73 | 43.45% |
|  | Bucca | 39 | 23.21% |
|  | Gingiva | 37 | 22.02% |
|  | Others | 19 | 11.31% |
| Differentiation | High | 103 | 61.31% |
|  | Medium/Low | 65 | 38.69% |
| T stage | T1/T2 | 102 | 60.71% |
|  | T3/T4 | 66 | 39.29% |
| Nodal invasion | Negative | 90 | 53.57% |
|  | Positive | 78 | 46.43% |
