# Supplementary material for: Prognostic value of tertiary lymphoid structure and tumour infiltrating lymphocytes in oral squamous cell carcinoma
Source: Int J Oral Sci. 2020 Sep 15;12:24. doi: 10.1038/s41368-020-00092-3 (PMC7493903; doi:10.1038/s41368-020-00092-3)
Supplement: Supplementary file 2 — Table S2 [file 41368_2020_92_MOESM2_ESM.docx]

**Table S2.** Univariate survival analysis of clinicopathological parameters

| Variables |  | Number (*n*=168) | 5-year OS (%) | *P* Value | 5-year RFS (%) | *P* Value |
| --- | --- | --- | --- | --- | --- | --- |
| Gender | Male | 120 | 62.5 | 0.371 | 70.0 | 0.895 |
|  | Female | 48 | 70.8 |  | 70.8 |  |
| Age | 57 | 79 | 60.8 | 0.335 | 72.2 | 0.600 |
|  | > 57 | 89 | 68.5 |  | 68.5 |  |
| Smoking | Current | 84 | 60.7 | 0.251 | 67.9 | 0.425 |
|  | Never or Former | 84 | 69.0 |  | 72.6 |  |
| Alcohol | Current | 77 | 63.7 | 0.596 | 68.1 | 0.438 |
|  | Never or Former | 91 | 66.2 |  | 72.7 |  |
| Tumor site | Tongue | 73 | 68.5 | 0.779 | 75.3 | 0.300 |
|  | Bucca | 39 | 59.0 |  | 61.5 |  |
|  | Gingiva | 37 | 67.6 |  | 75.7 |  |
|  | Others | 19 | 57.9 |  | 57.9 |  |
| Differentiation | High | 103 | 71.8 | 0.010* | 75.7 | 0.032* |
|  | Medium/Low | 65 | 53.8 |  | 61.5 |  |
| T stage | T1/T2 | 102 | 73.5 | 0.001* | 82.4 | <0.001* |
|  | T3/T4 | 66 | 51.5 |  | 51.5 |  |
| Nodal invasion | Negative | 90 | 80.0 | <0.001* | 77.8 | 0.017* |
|  | Positive | 78 | 47.4 |  | 61.5 |  |
| TLS | Positive | 45 | 88.9 | <0.001* | 88.9 | 0.002* |
|  | Negative | 123 | 56.1 |  | 63.4 |  |
| CD8 | Low | 83 | 48.2 | <0.001* | 61.2 | 0.009* |
|  | High | 85 | 81.9 |  | 79.5 |  |
| CD57 | Low | 97 | 52.0 | <0.001* | 63.3 | 0.016* |
|  | High | 71 | 82.9 |  | 80.0 |  |

TLS, tertiary lymphoid structure; ** P*<0.05.
