# Supplementary material for: Prognostic value of tertiary lymphoid structure and tumour infiltrating lymphocytes in oral squamous cell carcinoma
Source: Int J Oral Sci. 2020 Sep 15;12:24. doi: 10.1038/s41368-020-00092-3 (PMC7493903; doi:10.1038/s41368-020-00092-3)
Supplement: Supplementary file 3 — Table S3 [file 41368_2020_92_MOESM3_ESM.docx]

**Table S3.** Univariate and multivariate 5-year overall survival analysis of the TLS-positive patients

| Variables |  | Number (*n*=45) | 5-year OS  (%) | HR (95% *CI*) | *P* |
| --- | --- | --- | --- | --- | --- |
| Differentiation | High | 32 | 90.6 | Referent | 0.94 |
|  | Medium/Low | 13 | 84.6 | 0.916 (0.0-93-9.006) |  |
| T stage | T1/T2 | 32 | 87.5 | Referent | 0.729 |
|  | T3/T4 | 13 | 92.3 | 0.566 (0.023-14.054) |  |
| Nodal invasion | Negative | 31 | 96.8 | Referent | 0.030* |
|  | Positive | 14 | 71.4 | 16.361 (1.314-203.793) |  |
| Subtype of TLS | Mature TLS | 17 | 94.1 | Referent | 0.887 |
|  | Immature TLS | 28 | 85.7 | 0.816 (0.05-13.334) |  |

TLS, tertiary lymphoid structure; OS, overall survival; ** P*<0.05.
