# Supplementary material for: Prognostic value of tertiary lymphoid structure and tumour infiltrating lymphocytes in oral squamous cell carcinoma
Source: Int J Oral Sci. 2020 Sep 15;12:24. doi: 10.1038/s41368-020-00092-3 (PMC7493903; doi:10.1038/s41368-020-00092-3)
Supplement: Supplementary file 4 — Table S4 [file 41368_2020_92_MOESM4_ESM.docx]

**Table S4**. The association between TIL and TLS

| TIL | | TLS Grade | | | *P* |
| --- | --- | --- | --- | --- | --- |
|  |  | Grade 0 | Grade 1 | Grade 2 |  |
| CD8 | High | 46 | 21 | 18 | <0.001* |
|  | Low | 77 | 4 | 2 |  |
| CD57 | High | 40 | 15 | 16 | <0.001* |
|  | Low | 83 | 10 | 4 |  |

TIL, Tumor infiltrating lymphocyte; TLS, tertiary lymphoid structure; ** P*<0.05.
