# Supplementary material for: Prognostic value of tertiary lymphoid structure and tumour infiltrating lymphocytes in oral squamous cell carcinoma
Source: Int J Oral Sci. 2020 Sep 15;12:24. doi: 10.1038/s41368-020-00092-3 (PMC7493903; doi:10.1038/s41368-020-00092-3)
Supplement: Supplementary file 5 — Table S5 [file 41368_2020_92_MOESM5_ESM.docx]

**Table S5**. Summary of the OS predictive accuracy of TLS and TIL

| Predictive factors | AUC | SE | *P* Value | 95% *CI* | |
| --- | --- | --- | --- | --- | --- |
| T stage | 0.615 | 0.046 | 0.014* | 0.525 | 0.705 |
| Nodal invasion | 0.678 | 0.044 | <0.000* | 0.592 | 0.763 |
| TLS | 0.641 | 0.043 | 0.003* | 0.558 | 0.725 |
| CD8 | 0.682 | 0.042 | <0.000* | 0.599 | 0.765 |
| CD57 | 0.656 | 0.043 | 0.001* | 0.571 | 0.741 |
| All^a^ | 0.730 | 0.038 | <0.000* | 0.654 | 0.805 |

a, TLS+CD8+CD57; SE, standard error; 95% *CI*, 95% confidence interval; ** P*<0.05.
